# Susac's Syndrome: A Case with Unusual Cardiac Vestibular and Imaging Manifestations

**DOI:** 10.1155/2015/419408

**Published:** 2015-11-25

**Authors:** Yaron River, Avi Shupak, Beatrice Tiosano, Vika Danilov, Itzhak Braverman

**Affiliations:** ^1^Department of Neurology, Hillel Yaffe Medical Center, P.O. Box 169, 38100 Hadera, Israel; ^2^Unit of Otoneurology, Lin Medical Center, Haifa, Israel; ^3^Department of Otolaryngology, Head and Neck Surgery, Carmel Medical Center, Haifa, Israel; ^4^The Bruce Rappaport Faculty of Medicine, Technion-Israel Institute of Technology, Haifa, Israel; ^5^Department of Ophthalmology, Hillel Yaffe Medical Center, Hadera, Israel; ^6^Unit of Otolaryngology, Head and Neck Surgery, Hillel Yaffe Medical Center, Hadera, Israel

## Abstract

Susac's syndrome (SS) is a disease of the microvasculature of the retina, brain, and inner ear. We describe a patient with unusual manifestations of SS with possible involvement of the brainstem, cardiac arrhythmia, and MRI findings lacking the characteristic lesions found in Susac's syndrome.

## 1. Introduction

Susac's syndrome (SS) is the triad of encephalopathy, retinal artery branch occlusions, and hearing loss. The pathogenesis of SS is unknown; however, recent findings suggest that it is underlain by a distinct autoimmune endotheliopathy syndrome associated with anti-endothelial antibodies [1]. During the last three decades, more than 300 cases have been published [2]. Women are more commonly affected than men with a ratio of 3.5/1; the mean age of onset is 31. Headache may be the presenting feature. Multifocal neurological signs and symptoms, psychiatric disturbances, cognitive changes, memory loss, and confusion occur in 88% of the patients, retinal involvement occurs in 46% of the patients, and cochlear involvement occurs in 52% of the patients. Brain MRI may show a distinctive white matter changes that affect the supratentorial white matter particularly the corpus callosum, infratentorial white matter, cortical deep gray, and leptomeningeal involvement. SS requires treatment with immunosuppressants: steroids, cyclophosphamide, and intravenous immunoglobulin, usually in combination [2–4]. We describe a case of SS with several unusual features: cardiac involvement with persistent bradycardia, central vestibular involvement with upbeat nystagmus, and the late appearance of MRI white matter change.

## 2. Case Report

**Figure 1:**
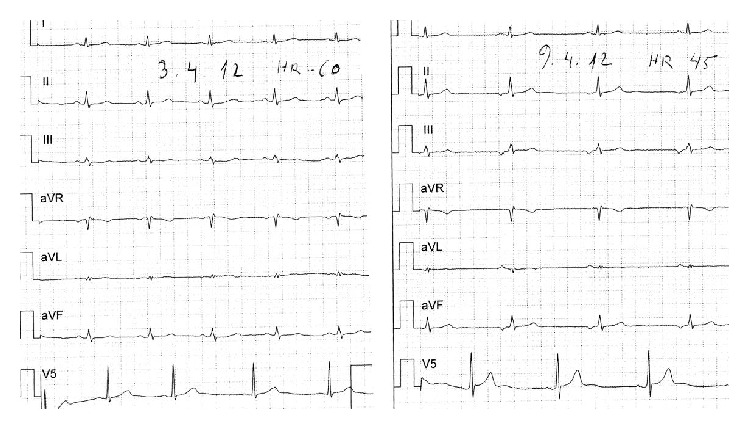
EKG: note the bradycardia and inverted P waves (II; III; AVF) 6 days following admission.

**Figure 2:**
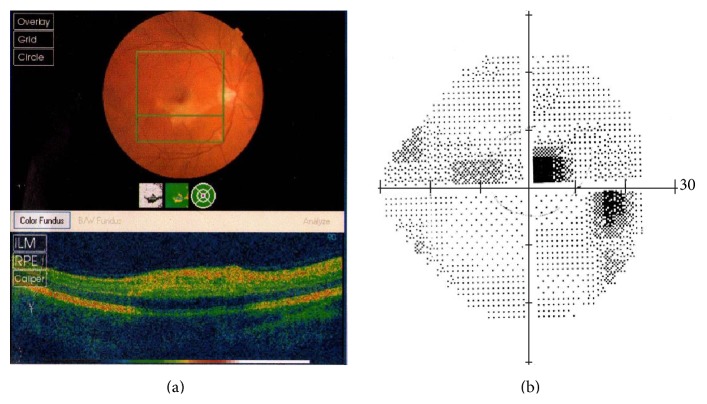
Left panel: ocular coherence tomography. Note the widening of the inner retinal layer signifying retinal edema three weeks following admission. Right panel: right eye visual field. Note the central scotoma.

A 38-year-old male was admitted to the Neurology Department at the Hillel Yaffe Medical Center due to severe headache of two days duration, dizziness, and confusion. Complete physical and neurological examination was normal. Brain CT scan was normal. CSF showed a protein level of 110 mg/dL, glucose level of 66 mg/dL, 2 lymphocytes, and negative test for oligoclonal bands. EEG showed slight symmetric slowing of background activity. The initial clinical diagnosis was encephalitis. He was treated with IV acyclovir. Two days after admission, a persistent bradycardia was noted with P wave EKG changes which lasted about two weeks (Figure 1). Cardiac transesophageal echocardiography was normal. Three days after admission, mild right lateralizing signs were noted, with very mild speech disturbance and reduced fluency. Brain CT angiography was normal. Five days later, the patient complained about blurred right eye vision. Fundoscopy did not show arterial wall plaques. Visual field examination showed a right eye small central scotoma and ocular coherence tomography study demonstrated inner retinal layer edema inferior to the fovea (Figure 2). Fluorescein angiography of the right eye disclosed central retinal artery branch occlusion. The patient was treated with Aspirin. At this point of time, workup also included negative studies for hypercoagulopathy, negative serological studies for antinuclear factor, anti DNA antibodies, and antineutrophil cytoplasmic antibodies. HLA-B51 antigen study was negative. Serologies for EBV, CMV, WNV, HSV-1, VZV, HIV, and* Enterovirus* were all negative.

**Figure 3:**
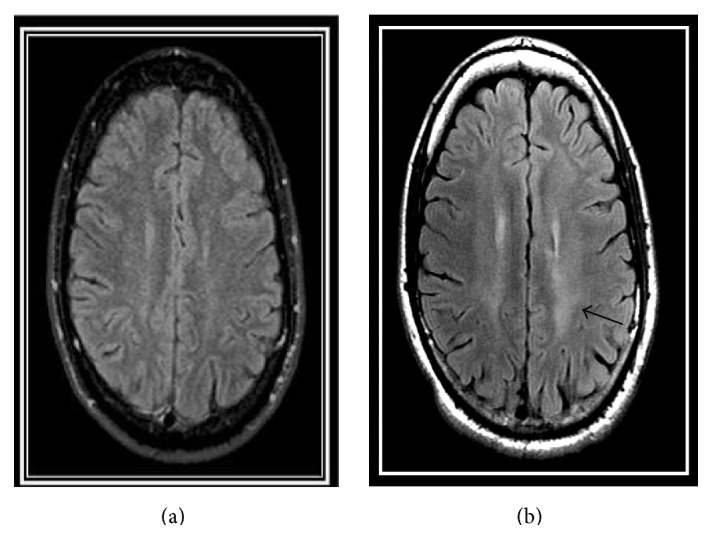
MRI-FLAIR protocol. Left: initial MRI, 04/12. Right: MRI following relapse 10/12. Note a diffuse hyperintense, ill-defined, left hemispheric centrum semiovale lesion superior to the lateral ventricles (black arrow).

Ten days after his admission, the patient reported intense right ear tinnitus with hearing loss, and this was followed, 24 hours later, by true vertigo and the illusion of whole body levitation. A formal audiogram 14 days after admission showed right flat sensorineural hearing loss (SNHL) of 50 Db threshold. A repeated audiogram three days later showed deterioration of the SNHL to 85 Db. Left side hearing was normal with SRT of 5 Db. Videonystagmography (VNG) showed upbeat vertical nystagmus, and right caloric weakness of 56% was demonstrated. Cochlear emissions were not demonstrated from both ears in TEOAE protocol. Brainstem evoked potential study showed normal I–IV wave patterns with interaural central conduction time asymmetry of 0.25 msec (longer right central conduction time). He was treated with Pulse methylprednisolone 1 gr per day for five days with concomitant IV immunoglobulin (IVIG) (2 gr/Kg). Brain MRI performed three weeks after admission was normal. Following treatment his condition remained stable with the disappearance of bradycardia and nystagmus. He was treated with a monthly dose of IVIG (0.4 gr/Kg). Six months later, a relapse with dizziness and blurred vision occurred. Examination showed left hand drift and dysdiadochokinesia with mild gait ataxia and positive left Fukuda test, repeated MRI showed an ill-defined T2 hyperintense signal of the left hemisphere without the typical MRI findings of Susac's syndrome (Figure 3). The steroid dose was increased to prednisone 60 mg/day with the resolution of symptoms in four days. Monthly 0.4 gr/Kg IV immunoglobulin was continued for 14 months after the initial presentation.

20 months after the initial presentation, a relapse occurred with visual disturbance without other manifestations. Fluorescein angiography disclosed left eye central retinal artery branch occlusion. Brain MRI was normal. He was treated with pulse Methylprednisolone 1 gr per day for five days with concomitant 2 gr/Kg IV immunoglobulin.

His condition remained stable for further 8 months with monthly IV immunoglobulin treatment.

28 months after presentation the patient developed right eye blurred vision. Workup was positive for a new right eye lower nasal scotoma with concomitant right eye temporal retina ischemic changes. He was treated with pulse Methylprednisolone 1 gr per day for five days and then he was started on Azathioprine 50 mg per day. Azathioprine was stopped after a few weeks because of severe diarrhea and the patient was started on Mycophenolate 2 gr per day with concomitant monthly 0.4 gr/Kg IV immunoglobulin. The patient has been clinically stable for the last 13 months with monthly IV immunoglobulin and daily Mycophenolate treatment.

## 3. Discussion

We describe a patient with the triad of encephalopathy with right lateralizing signs, retinal artery branch occlusion, and right hearing loss. This is a case with the classic triad of Susac's syndrome. However, the patient presented several unusual features. For two weeks, the patient had persistent bradycardia. The inverted P wave suggests an ectopic atrial rhythm not originating from the sinus node possibly due to sinus arrest. One explanation for the bradycardia is cardiac involvement particularly of the conduction system. Disorders with a similar autoimmune endotheliopathy such as dermatomyositis have approximately 70% of patients with evidence of cardiac damage; in one-third of cases, this affects principally or solely the conduction tissue [5, 6]. The possibility of intrinsic cardiac involvement does not explain other unusual features of this case particularly the upbeat nystagmus. It is our view that a more plausible explanation for the upbeat nystagmus and persistent bradycardia is the assumption that our patient had a medulla oblongata lesion which was not demonstrated by MRI.

Damage to the central projections of the anterior semicircular canals, the ventral tegmental tract, which tends to deviate the eyes superiorly, has been suggested to explain upbeat nystagmus [7]. In particular, posterior medullary lesions, in the vicinity of the nucleus of Roller, could disrupt a medullary inhibitory input to the flocculus. This in turn could bring about overinhibition of the superior vestibular nucleus and downward deviation of the eyes with “compensatory” upbeat nystagmus [8]. The nucleus of Roller is part of a group of nuclei anterior and lateral to the hypoglossal nucleus which are closely located to the nucleus solitarius. The nucleus and tractus solitarius is a major hindbrain area involved in cardiovascular regulation. It receives primary afferent fibers from peripheral baroreceptors and chemoreceptors [9]. Animal model studies and clinical human data suggest that lesions of the medullary tegmentum, particularly lesions that involve the nucleus solitarius, are capable of inducing prolonged bradycardia with sinus arrest. Bradycardia in those cases is supposedly a result of central imbalance between the sympathetic and parasympathetic systems [9–11]. Two consecutive MRI scans failed to show a structural brain abnormality and in particular a brainstem lesion in spite of the definite neurological involvement. Left hemisphere periventricular white matter lesion was noted only in the third MRI scan, following a relapse, half a year after the diagnosis. Rennebohm et al. argued that the diagnosis of SS can be made when only the encephalopathy and pathognomonic MRI lesions are present; the hearing loss and retinal artery branch occlusion need not be present [12]. However, the lack of MRI findings does not exclude the diagnosis of SS which is essentially a clinical diagnosis.

The nontypical findings on the third MRI, the lack of any abnormal findings in the initial MRI scans, and in specific the lack of corpus callosum lesions, which are the characteristic lesions found in SS, raise few likely explanations. (a) A subset of patients with SS run a relatively benign course with few MRI lesions. (b) SS might be a more heterogeneous syndrome than previously recognized. (c) The utilization of special MRI techniques, not performed in this case, might unravel lesions not recognized by conventional MRI. For instance, abnormal prefrontal diffusion tensor imaging scans are better correlated with the severity of encephalopathy than by the mostly sparse white matter abnormalities seen on conventional MRI [12].

This case with unusual MRI findings, the appearance of bradycardia, the lack of arterial retinal plaques raises an unresolved question about the gold standard for the diagnosis of Susac's syndrome. What actually defines this syndrome when some of the key findings are not present? The discovery of anti-endothelial antibodies which provide a pathophysiological putative mechanism will perhaps allow us to differentiate Susac's disease with a seropositive clinical picture from Susac's syndrome with a seronegative clinical picture.

In conclusion, (1) transient bradycardia could be a manifestation of SS due to brainstem involvement.

(2) Upbeat nystagmus in this case is not the result of labyrinthine or 8th cranial nerve damage. It is more likely a central phenomenon which is underlain by the disruption of central vestibular pathways.

(3) Initial MRI could be normal in patients with SS. However, repeated scans will probably disclose white matter lesions, not necessarily the typical changes described in the central part of the corpus callosum [2].

(4) Patients with SS might have repeated relapses in spite of treatment with steroids and IV immunoglobulin. These patients might require a more aggressive treatment with combination of IV immunoglobulin and an immunosuppressant drug.
